# Drug resistance and population structure of *Plasmodium falciparum* and *Plasmodium vivax* in the Peruvian Amazon

**DOI:** 10.1038/s41598-022-21028-3

**Published:** 2022-10-01

**Authors:** Fredy E. Villena, Juan F. Sanchez, Oscar Nolasco, Greys Braga, Leonila Ricopa, Keare Barazorda, Carola J. Salas, Carmen Lucas, Stephen E. Lizewski, Christie A. Joya, Dionicia Gamboa, Christopher Delgado-Ratto, Hugo O. Valdivia

**Affiliations:** 1 Vysnova, Lima, Peru; 2 Department of Parasitology, U.S. Naval Medical Research Unit No. 6 (NAMRU-6), Lima, Peru; 3 Instituto de Medicina Tropical Alexander Von Humboldt, Universidad Peruana Cayetano Heredia, Lima, 31 Peru; 4 Departamento de Ciencias Celulares y Moleculares, Facultad de Ciencias y Filosofía, Universidad Peruana Cayetano Heredia, Lima, 31 Peru; 5 Malaria Research Group (MaRCH), Global Health Institute, University of Antwerp, 2610 Antwerp, Belgium

**Keywords:** Parasite genomics, Population genetics

## Abstract

Malaria is a major health problem in Peru despite substantial progress achieved by the ongoing malaria elimination program. This study explored the population genetics of 63 *Plasmodium falciparum* and 170 *P. vivax* cases collected in the Peruvian Amazon Basin between 2015 and 2019. Microscopy and PCR were used for malaria detection and positive samples were genotyped at neutral and drug resistance-associated regions. The *P. falciparum* population exhibited a low nucleotide diversity (π = 0.02) whereas the *P. vivax* population presented a higher genetic diversity (π = 0.34). All *P. falciparum* samples (n = 63) carried chloroquine (CQ) resistant mutations on *Pfcrt*. Most *P. falciparum* samples (53 out of 54) carried sulfadoxine (SD) resistant mutations on *Pfdhfr* and *Pfdhps*. No evidence was found of artemisinin resistance mutations on *kelch13*. Population structure showed that a single cluster accounted for 93.4% of the *P. falciparum* samples whereas three clusters were found for *P. vivax*. Our study shows a low genetic diversity for both species with significant differences in genetic sub-structuring. The high prevalence of CQ-resistance mutations could be a result of indirect selection pressures driven by the *P. vivax* treatment scheme. These results could be useful for public health authorities to safeguard the progress that Peru has achieved towards malaria elimination.

## Introduction

Malaria remains a major health problem in tropical and subtropical regions causing more than 241 million cases and over 627,000 deaths during 2020^1^. Despite major progress achieved towards malaria elimination in the Americas, this disease still puts at risk of infection to nearly 138 million people leading to 889,000 new cases each year. Furthermore, up to 90% of these cases occur in Amazonian regions of Venezuela, Brazil, Colombia and Peru^2^.

In Peru, the Amazonian region of Loreto accounts for 84% of the 15,519 malaria cases reported in 2020 with most of those caused by *Plasmodium vivax* followed by *P. falciparum* (ratio Pv/Pf = 4)^3^. Malaria transmission is perennial in this region, with seasonal increases of cases between February and July^4^.

The malaria treatment policy for uncomplicated *P. falciparum* infections consists of mefloquine (MQ, 12.5 mg/kg/day for 2 days) plus artesunate (AS, 4 mg/kg/day for 3 days). In contrast, treatment for *P. vivax* comprises chloroquine for three days (CQ, 10 mg/kg/day the first two days and 5 mg/kg/day on the third day) plus primaquine (PQ, 0.5 mg/kg/day for 7 days). The change to MQ-AS was implemented nearly two decades ago due to the widespread prevalence of CQ resistance in *P. falciparum*^5^.

In Peru, malaria incidence has presented several fluctuations over the last two decades. One of these fluctuations occurred during the implementation of the Global Fund's Malaria Project “PAMAFRO” (2005–2011)^6^. This project aimed to increase access and quality to microscopy diagnosis, treatment and capacity building. Due to PAMAFRO activities, the number of malaria cases reported per year in Peru declined from 87,805 cases in 2005 to 23,060 cases in 2011. The prevalence reduction was higher for *P. falciparum* (85%) than *P. vivax* (63%)^7^. However, after PAMAFRO ended, interventions were not sustained, and malaria cases substantially increased from 23,060 cases in 2011 to 56,530 cases in 2016^7^.

In 2017, the Peruvian government launched a malaria elimination initiative called *Plan Malaria Cero* (PMC) aiming to eliminate malaria within 25 years from the Loreto region. PMC has three phases: Phase I, malaria control; II, towards malaria elimination; and III, residual malaria elimination^8^. During Phase I, the malaria cases in 2020 were reduced by more than 75% compared to 2017 (15,519 vs 55,227, respectively). PMC's phase II has ahead multiple challenges, e.g., increasing focal cases distribution, transmission shift to rural and remote settings, an increment in the prevalence of asymptomatic and submicroscopic cases among others^7^. Thanks to the successful control outcome achieved by PMC, last January 2022, the government extended it to a national level initiative for malaria elimination for 2030.

As PMC's elimination efforts continue, they will impact circulating parasite populations as previously shown^9–12^. In this regard, malaria population genetic studies conducted in the Peruvian Amazon showed a higher population heterogeneity in *P. vivax* transmission than in *P. falciparum*^13^*.* Furthermore, communities with variable connectedness showed high diversity and polyclonal infections ranging between 44 and 70%^14^ compared to isolated communities characterized by limited genetic diversity and low frequency of polyclonal infections (14–19.7%)^15–17^. Regarding *P. falciparum,* parasite populations probably expanded from bottlenecked populations after malaria eradication efforts from 1966 to 1989^10^. These Peruvian *P. falciparum* populations consisted of at least five clonal lineages and presented a low proportion of polyclonal infections^10^.

*Plasmodium* genomic plasticity is an important threat for malaria elimination as it can lead to the relatively rapid development of drug resistance against antimalarials^18^. In addition, migration, climate change and human-driven activities can enhance drug resistance spread or introduce new strains across regions^11,12,19^. Therefore, it is key to implement stronger surveillance platforms in endemic settings that can allow for early detection of drug resistance or emergence of virulent strains. This is particularly important in Peru due to the lack of data after the implementation of new malaria elimination policies in 2001.

This study, explored the population genetics and molecular surveillance of mutations associated with drug-resistance in *P. falciparum* and *P. vivax* parasites circulating in the city of Iquitos and surrounding communities of the Loreto region, Peru, between 2015 and 2019. The information provided by this study will contribute with relevant information to guide malaria elimination efforts in Peru.

## Materials and methods

### Study sites, sample selection

**Figure 1:**
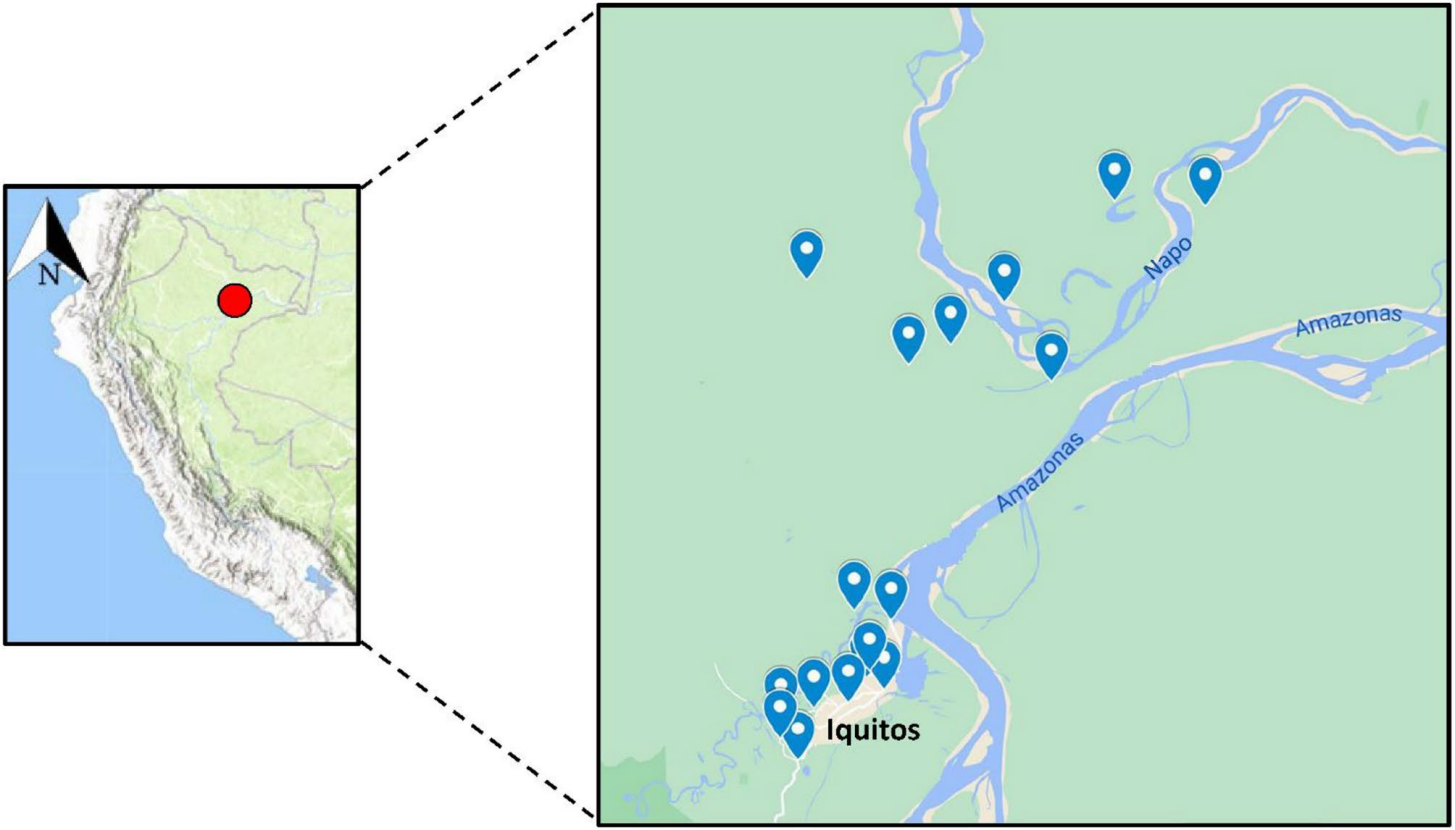
Map of the study sites located in the region of Loreto. The inset shows the location of the field sites in and around Iquitos city depicted in blue marks. The map was created using ArcGIS online (ESRI Inc. Redlands, CA, USA. https://www.esri.com/) using open data obtained from GADM database of Global Administrative Areas, version 3.6. www.gadm.org.

The Loreto region is located in the Northeast Peruvian Amazon and is characterized by an equatorial climate with annual temperatures between 24 and 33 °C. Blood spot samples for this study were collected from sixteen study sites in Iquitos (Loreto's capital city) and surrounding communities as part of two surveillance projects conducted between 2015 and 2019 (Fig. 1). The sample collection was conducted by U.S. Naval Medical Research Unit 6 (NAMRU-6) and Universidad Peruana Cayetano Heredia (UPCH).

The NAMRU-6 project was a passive surveillance study that enrolled people > 1-year-old with of fever or history of fever during the previous 72 h in different health centers across the city. The UPCH project was an active surveillance study carried out in 2018 to screen high-risk malaria populations in various communities. Inhabitants from Quistococha, Santo Tomas and Rumococha communities were enrolled in April from Gamitanacocha, Libertad, 1 de Enero, Salvador, Lago Yuracyacu, Puerto Alegre, and Urcomiraño communities in July 2018.

### Ethics

The samples tested for this study were selected from two sources: i) the NAMRU-6 project which was approved by the Institutional Review Board of the U.S Naval Medical Research Unit 6 (NAMRU-6) in compliance with all applicable federal regulations governing the protection of human subjects (protocol NMRCD.2007.0004) and ii) The UPCH projects which was approved by the Institutional Ethical Review Board of the Universidad Peruana Cayetano Heredia (protocols SIDISI 101645/2017 and SIDISI 101518/2018). Informed consent was obtained from all participants and/or their legal guardians. All methods were performed in accordance with the relevant guidelines and regulations.

### Sample processing and malaria diagnostics

Two thin and thick smears were prepared for each participant and stained with Giemsa. Slides were read by two microscopists whereas a third microscopist reviewed slides with discordant results.

DNA from whole blood was extracted using the Qiagen DNA extraction kit according to the manufacturer’s protocol. Malaria-positive cases were detected by Malachite green LAMP or real-time PCR as previously described^20–22^. For MG-LAMP, the reaction was performed in a 20 µL reaction volume that contained 5 µL of template DNA in 2X in-house reaction buffer (40 mM Tris–HCl pH 8.8, 20 mM KCl, 16 mM MgSO_4_, 20 mM (NH_4_)_2_SO_4_, 0.2% Tween -20, 1.6 M Betaine, 2 mM of dNTP's each), 0.25µL of 1:400 SYTO 9 dye, 8 units of Bst Polymerase (New England Biolabs, Ipswich, MA) and 0.004% Malachite Green dye. DNA amplification was carried out at 63 °C for 60 min and two independent readers visually inspected the results after 15 min post amplification. For the real-time PCR, the reaction was performed in a 25 µL reaction volume that contained 5 µL of template DNA in a 1X master mix of PerfeCTa SYBR® Green Fastmix, 0.3 μM of each primer PL1473F18 5′-TAACGAACGAGATCTTAA-3′ and PL1679R18 5′-GTTCCTCTAAGAAGCTTT-3′. The real-time PCR conditions consisted of an initial denaturation step at 95 °C for 2 min, followed by 45 cycles of 20 s at 95 °C, 20 s at 50 °C, and 20 s at 68 °C.

### Sequencing and SNP genotyping

Blood spots from malaria-positive samples were sent to the Welcome Sanger Institute for sequencing, evaluation of drug resistance markers and barcodes generation^23^. A selective whole genome amplification was used to enrich target regions of DNA by multiplex PCR. Additionally, an extra round of PCR was done to incorporate adapters on both *P. falciparum* and *P. vivax* and subsequently, these were pooled and sequenced on Illumina MiSeq sequencer. Finally, reads from each sample were aligned onto the *P. falciparum* 3D7 or *P. vivax* P01 reference sequences.

Sample barcodes were generated for *P. falciparum* (101 SNPs) and *P. vivax* (38 SNPs). The barcodes consist of biallelic SNPs selected from the malaria genome variation database based on their usefulness to evaluate parasite interrelationship. Barcodes are distributed across the *P. vivax* and *P. falciparum* genomes and have not been associated to drug resistance^23,24^.

### Drug resistance markers

In *P. vivax*, three drug-resistance markers were evaluated: *Pvdhfr* (positions 57, 58, 61 and 117) and *Pvdhps* (380, 382, 383, 385 and 553) associated with sulfadoxine-pyrimethamine resistance and *Pvmdr1* (976) that is putatively associated with CQ resistance^25–27^. In the case of *P. falciparum,* mutations in nine markers were evaluated: *arps10* (positions 127 and 128), *ferredoxin* (193), *Pfcrt* (326 and 356)*, k13* (BTB/POZ and propeller) and *Pfmdr2* (484) associated with artemisinin resistance^28,29^, *Pfdhfr* (51, 59, 108 and 164) and *Pfdhps* (436, 437, 540, 581 and 613) associated with SP resistance^30–33^, the exonuclease gene, *Pfexo* (415) associated with piperaquine resistance, *Pfcrt* (72, 73, 74, 75 and 76) associated with chloroquine resistance and *Pfmdr1* (86, 184 and 1246) associated with CQ, amodiaquine, lumefantrine and MQ resistance^34–39^. Markers were combined for each sample in order to assess the prevalence of common drug resistance haplotypes.

### Data analysis, multiplicity of infection and population diversity

Complexity of infection (COI) was analyzed by a Markov Chain Monte Carlo (MCMC) method using COIL and Real McCOIL which estimate the proportion of heterozygous calls^40,41^.

Barcodes were subsequently filtered using the R package *poppr*^42^ to exclude samples with > 20% missing calls and positions with missing calls in > 20% of the samples. The resulting barcoding data was used to estimate the population barcode diversity (π) using 10,000 iterations of nonparametric bootstrapping^24^. Moreover, an Analysis of Molecular Variance (AMOVA) was performed to estimate the strength of genetic differentiation among Plasmodium populations using Arlequin 3.5 by a locus-by-locus analysis with 1000 permutations^43^.

### Phylogenetic and Principal Component Analysis

Phylogenetic reconstruction was carried out using only monoclonal samples using a maximum likelihood approach in PhyML v3.0^44^ with 1000 bootstrap and under the best-fit model defined by Bayesian information criterion obtained from jModelTest 2.1.5^45^. The *P. falciparum* 3D7 and *P. vivax* P01 strains were used to root the phylogenetic trees visualized in iTOL^46^.

In addition, principal components analysis (PCA) was carried out on monoclonal samples using the R package *adegenet*^47^ to estimate the parasite population structure. To evaluate the relationship among *P. falciparum* haplotypes, a phylogenetic network was built adding control samples from Africa (n = 11), South East Asia (n = 10) and South America (n = 15) on PopART^48^ using the median-joining algorithm. This method was also employed for *P. vivax* adding control samples from Colombia (n = 26) and Honduras (n = 18).

### Epidemiological analysis

Clinical and epidemiological data were analyzed in Stata 16. The potential association of clinical data, disease severity, sociodemographic data and laboratory results with specific *P. vivax* parasite populations was analyzed using Fisher's exact test or Kruskal–Wallis test.

## Results

### Sample collection and sociodemographic data

A total of 67 participants positive for *P. falciparum* and 170 positives for *P. vivax* were enrolled in both studies (Table 1). Up to 61.5% (217 out of 237) of all participants were enrolled in 2018. The median participant age was 27 years old (IQR: 18–46 y.o) and mainly males (66.2%) for *P. vivax* and 37 years old (IQR: 22–58 y.o) and 62.7% males in *P. falciparum*.

**Table 1:** Sociodemographic and household characteristics of enrolled participants.

Characteristics	Vivax malaria	Falciparum malaria
	(n = 170)	(n = 67)
**Socio-demographic**		
Age (in years)*	28.5 (17–45)	37 (22–58)
Male	113 (66.47)	42 (62.69)
**Occupation**		
Agriculture	24 (14.20)	21 (31.34)
Housewife	28 (16.57)	14 (20.90)
Student	43 (25.44)	10 (14.93)
Logger	1( 0.59)	0 (0.00)
Health worker	1 (0.59)	1 (1.49)
Other	73 (42.90)	21 (31.34)
**Health facility (HF)**		
Hospital de Apoyo	9 (5.29)	3 (4.48)
Bellavista-Nanay HF	41 (24.12)	9 (13.43)
Moronacocha HF	30 (17.65)	20 (29.85)
Padrecocha HF	32 (18.82)	14 (20.90)
Quistococha HF	1 (0.59)	0 (0.00)
Rumococha HF	13 (7.65)	0 (0.00)
San Juan HF	3 (1.76)	5 (7.46)
Santa Clara HF	30 (17.65)	10 (14.93)
Santo Tomas HF	2 (1.18)	0 (0.00)
Tupac HF	9 (5.29)	1 (1.49)
Mazan HF	0 (0.0)	5 (7.46)
**Clinical**		
Headache	161 (94.71)	61 (91.04)
Malaise	149 (96.13)	60 (89.55)
Chills	150 (88.24)	58 (86.57)
Jaundice	0 (0.00)	28 (49.12)
Fever	162 (95.29)	61 (91.04)
Sweating	132 (85.16)	48 (71.64)
Diarrhea	15 (9.68)	7 (12.28)
Vomiting	14 (8.24)	10 (14.93)
Seizures	6 (3.87)	2 (3.57)
Breathing problems	36 (23.38)	15 (26.32)
Pregnancy	4 (7.02)**	1 (3.85)***
**Laboratory**		
Asexual parasitaemia determined by microscopy (par/uL)*	8,405.5 (3676–14,554)	2,913 (750–6877)
Asexual parasitaemia determined by microscopy (log par/uL)*	9.05 (8.24–9.60)	7.99 (7.19–8.87)
Sexual parasitaemia determined by microscopy (par/uL)*	116 (59–182)	0 (0–29)
Sexual parasitaemia determined by microscopy (Log par/uL)*	4.79 (4.08–5.56)	4.46 (4.07–5.66)****
**Epidemiology**		
Malaria episodes at last 10 years*	3 (1–5)	2 (0–4)
Malaria episodes at last year*	1 (0–2)	0 (0–1)
Relative with malaria at the last year	63 (44.68)	22 (38.60)

* Median (IQR)

**(n = 57),

***(n = 26),

****(n = 18).

Regarding households, 45.3% of the *P. vivax* and 38.6% of participants carrying *P. falciparum* reported having a family member with malaria in the last year. More than 50% of participants carrying *P. vivax* presented at least three malaria episodes in the previous 10 years whereas 50% of participants carrying *P. falciparum* presented at least two episodes during the same period.

The most frequent clinical symptoms in the study population were headache, malaise, chills, sweating and fever. There were not significant differences in clinical symptoms between people infected with *P. vivax* or with *P. falciparum.*

### Haplotypes and drug resistance polymorphisms

SNPs genotyping of *P. falciparum* showed that 100% of the samples (n = 52) carried the 184F/1246Y double *Pfmdr1* mutation. The SVMNT haplotype on *Pfcrt* was present in 98.4% of the samples (63/64). None of the samples presented parasite genetic background mutations on *Pfarp10*. One sample had a 617S mutation in *Pfk13* whereas the rest were wild-type.

Most *P. falciparum* samples (98%; 53/54) had quadruple mutations at *Pfdhfr* and *Pfdhps* (51I/108N + 437G/540E), which is a combination that has been associated with SP treatment failure^32^. In addition, our study did not find mutations on *PfEXO* associated with piperaquine resistance nor *FERREDOXIN* and *Pfmdr2* associated with artemisinin resistance (Tables S1 and S2)^29,33^. The most frequent haplotype for this species based on *Pfdhfr*, *Pfdhps, Pfcrt*, *PfEXO, Pfmdr1, Pfarps-10*, *FERREDOXIN* and *Pfmdr2* (**I**C**N**IS**GEG**A**S**VMN**TDL**EN**FY**VDDT) accounted for more than 57% of the samples (Table S2).

The 976F mutation on *Pvmdr1* was prevalent on 4.1% *P. vivax* infections (7 out of 170). For *Pvdhfr*, 0.6% cases (1/161) presented the 57L mutation, 97.5% (154/158) were mutants on position 58 (R, K or L), and 97% (165/170) presented the 117 N mutation with 48% (76 out of 158) harboring the double mutation S58R and S117N. For *Pvdhps*, 72.3% (123/170) of the infections presented the 383G mutation. All infections carried wildtype parasites for *Pvdhps* for positions 385, 553 (Tables S3 and S4). The most frequent *P. vivax* haplotype based on *Pvdhfr, Pvdhps* and *Pvmdr1* (F**K**T**N**E**CG**YAY) accounted for 31% of the samples and the next prevalent haplotypes accounted for 15,9% (F**R**T**N**E**CG**YAY) and 12.4% (F**R**T**N**ESAYAY) of the samples (Table S3).

### Population diversity and complexity of infection

All *P. falciparum* samples (n = 67) were monoclonal by COIL and real McCOIL methods. The barcode intrapopulation diversity for this species was π = 0.02 (95%CI: 0.007–0.039). In *P. vivax*, 10 out of 170 samples were polyclonal infections (10.6%) and the overall intrapopulation diversity was higher than *P. falciparum* with π = 0.34 (95%CI: 0.309–0.381).

### Population structure

Data cleaning resulted in 46 *P. falciparum* and 145 *P. vivax* samples passing the quality filters for population analysis and two loci in *P. falciparum* were removed. One sample (MDP4546) was excluded from DAPC due to being very different from the rest of *P. falciparum*.

**Figure 2:**
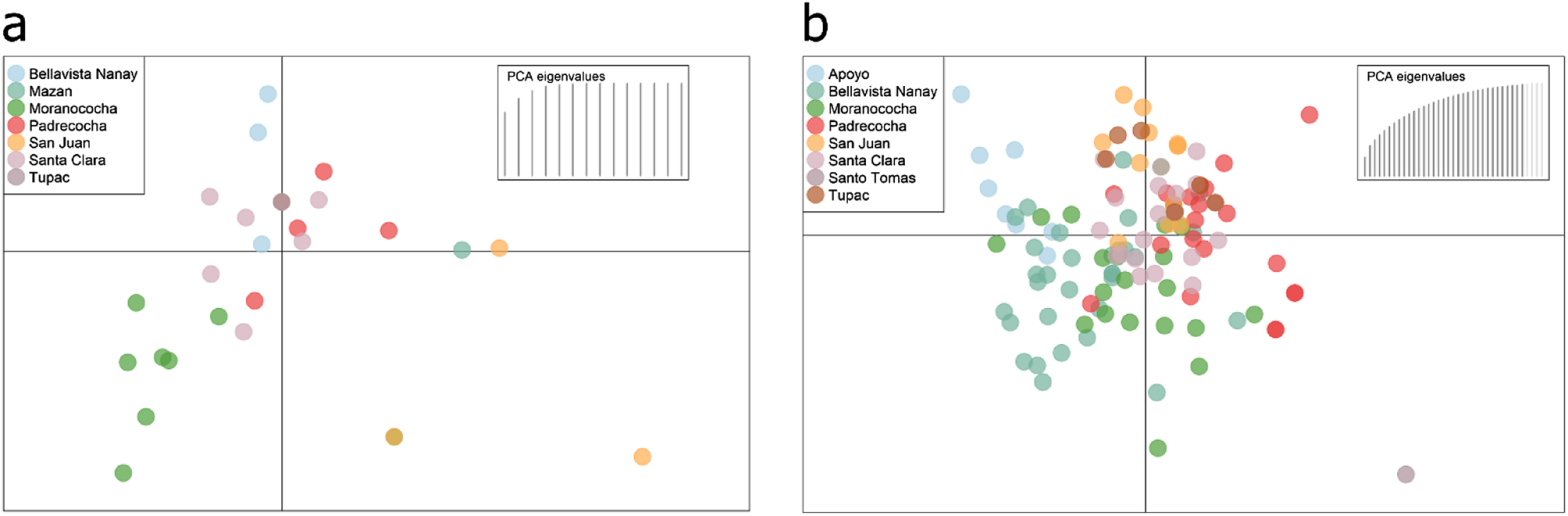
Discriminant analysis of principal components (DAPC) *P. falciparum* and *P. vivax*. The figure shows the population structure of 46 *P. falciparum* (**a**) and 145 *P. vivax* samples (**b**). The color scheme corresponds to each of the sites where samples were collected. DAPC shows the absence of clustering due to geographical origin.

DAPC on the rest of the samples did not show population sub-structuring related to geographic origin for both *P. vivax* (Fig. 2A) and *P. falciparum* (Fig. 2B). AMOVA's showed that 98% and 100% of the genetic variation relied within *P. vivax* and *P. falciparum’s* populations (p-value = 0.13 and 0.69, respectively) (Tables S5 and S6).

**Figure 3:**
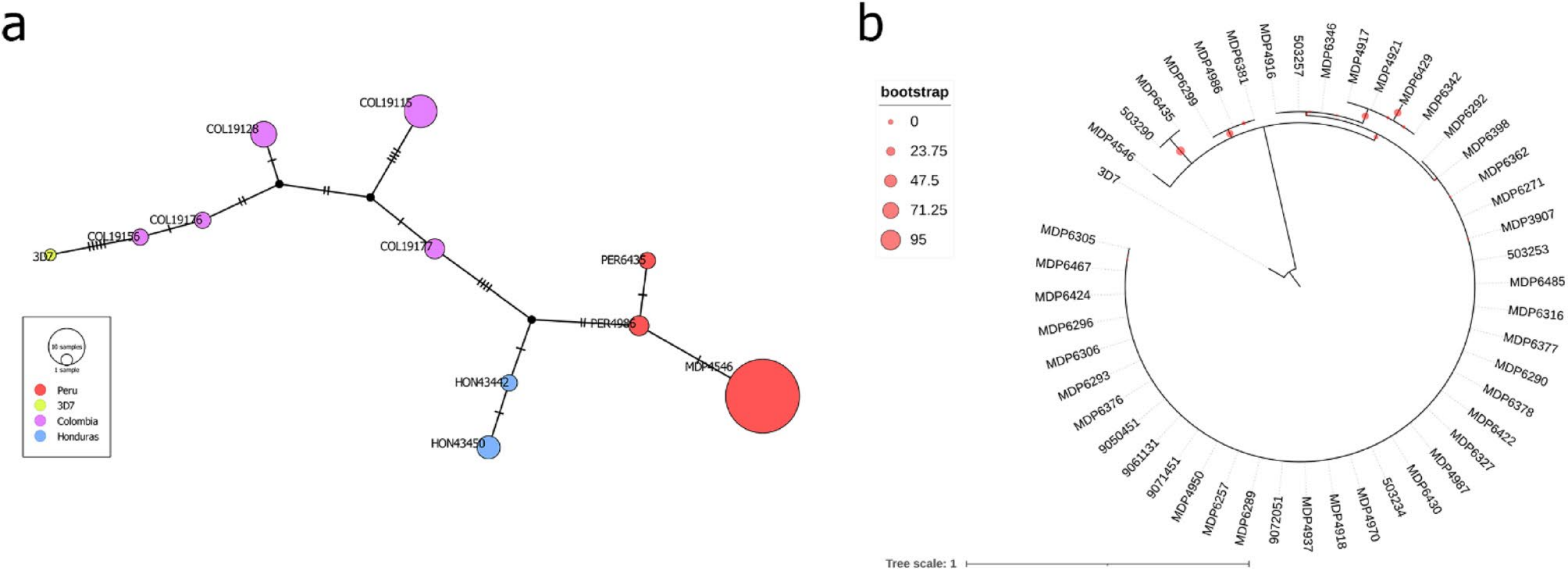
Phylogenetics results. (**a**) Median-joining network of Peruvian *P. falciparum* strains and control samples from South America (Honduras and Colombia). The network shows the distribution of lineages according to their barcode and the geographical clustering of *P. falciparum* lines. Each circle represents an independent haplotype, the lines connect nearby haplotypes and the cross line represents one non-synonymous mutation. Figure created in PopART^48^. (**b**) Maximum likelihood phylogenetic analysis of *P. falciparum* samples. Circles in tree nodes represent bootstrap support values, 3D7 denotes *P. falciparum* 3D7 strain. Figure generated in Figtree (http://tree.bio.ed.ac.uk/software/figtree/).

In *P. falciparum*, median-joining network showed an apparent clustering of parasites according to the country of isolation (Fig. 3A). Phylogenetic analyses showed that sample MDP4546 was distant from the rest of Peruvian strains (Fig. 3B).

**Figure 4:**
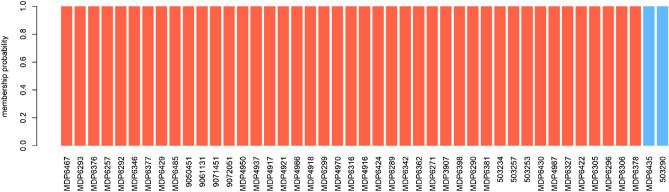
K-means clustering for *P. falciparum* samples. The y-axis denotes the membership probability of each sample to belong to a cluster whereas the color defines the two clusters that were identified for *P. falciparum*.

After removing sample MDP4546, K-means clustering showed two subpopulations with no evidence of admixed individuals (Fig. 4). One of these subpopulations accounted for 95.5% of all *P. falciparum* samples (43 out of 45 samples).

**Figure 5:**
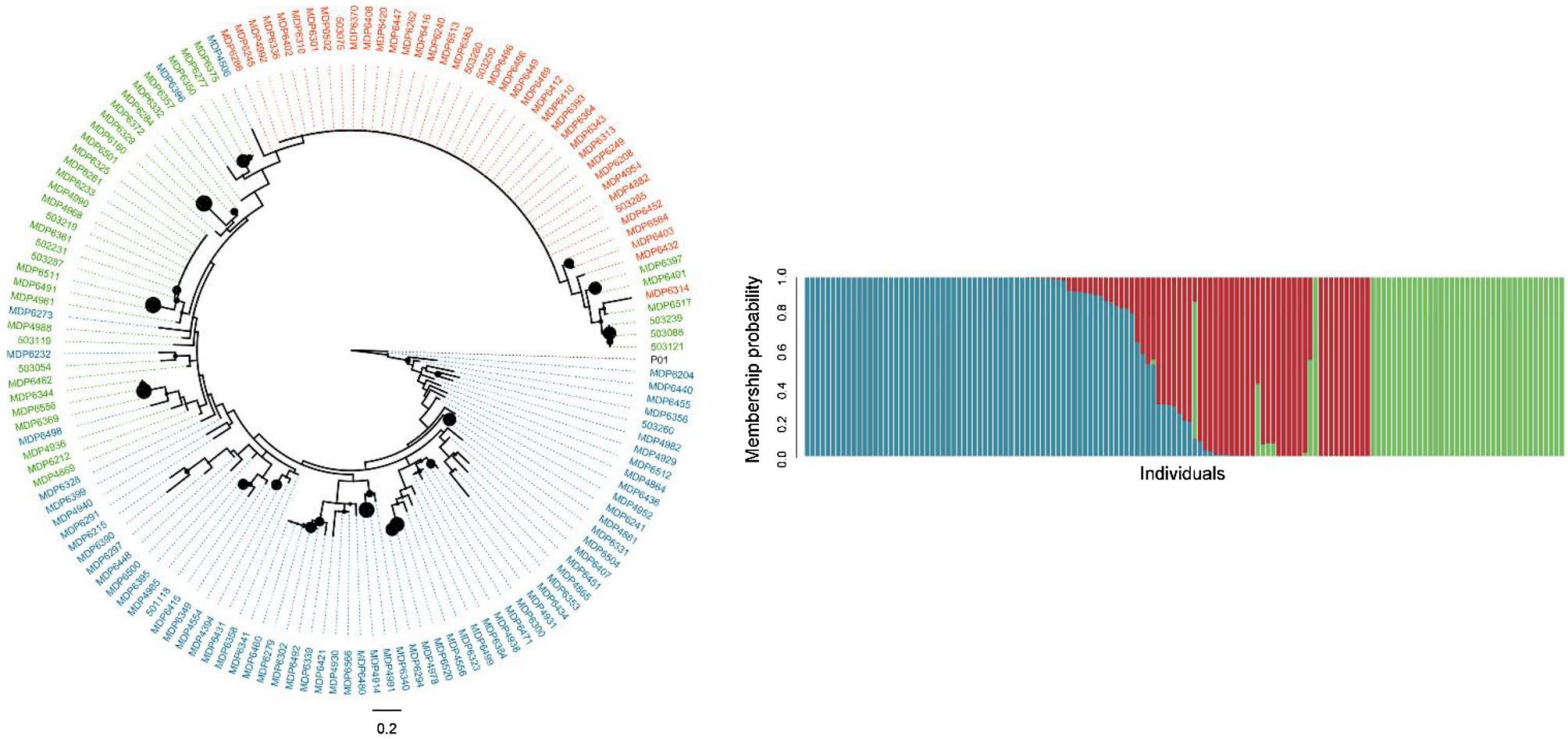
Phylogenetic relationship and population structure of *P. vivax.* Inset A shows the Phylogenetic three for *P. vivax*. Black colored circles denote bootstrap support > 700. Colored circles on tree tips match the three clusters defined by DPCA. P01 denotes the *Plasmodium vivax* P01 reference. Figure generated in Figtree (http://tree.bio.ed.ac.uk/software/figtree/). Inset B Shows a DAPC analysis with K-means clustering for *P. vivax***.** The y-axis denotes the membership probability of each sample to one of the three clusters defined by this analysis. Figure created musing the adegenet package^47^.

In the case of *P. vivax*, K-means clustering and phylogenetic analysis revealed the presence of three subpopulations with 21 admixed individuals (Fig. 5). The median-joining network for *P. vivax* showed that some samples from Colombia were closer to Peru than to the rest of samples from Colombia (Supplementary Fig. 1).

Malaria population structure matched with the drug resistance haplotypes for both *P. vivax* and *P. falciparum*. In this sense, most samples of the *P. falciparum* predominant cluster carried point mutations on genes associated with SP, MQ and CQ resistance.

In *P. vivax*, the three subpopulations matched with three haplotypes associated with SP resistance as previously shown. In this regard, significant differences were found between the three *P. vivax* genetic clusters for putative drug-resistance associated SNPs for positions *Pvdhfr*:58, *Pvmdr1*:976, *Pvdhps*:382 and *Pvdhps*:383 (Table 2) with populations 2 and 3 presenting higher prevalences for these SNPs than population 1.

**Table 2:** Differences in prevalence of putative drug resistant SNPs among *P. vivax* populations.

Genotype	Drug	Population 1	Population 2	Population 3	*p-value*
*Pvdhfr*:58R	SP (n, %)	2 (5.2%)	40 (65.5%)	21 (63.6%)	< 0.0001
*Pvmdr1*:976F	CQ (n, %)	0 (0%)	2 (3.2%)	4 (11.4%)	0.0501
*Pvdhps*:382A	SP (n, %)	1 (2.5%)	30 (49.2%)	4 (11.4%)	< 0.0001
*Pvdhps*:383G	SP (n, %)	38 (52%)	31 (100%)	31 (100%)	< 0.0001

## Discussion

In response to the malaria threat, Peru’s launched in 2017 a malaria elimination plan called Plan Malaria Cero ^8^, and in 2022 the program has expanded to a national level^49^. PMC advances caused significant malaria transmission disruptions and a potential parasite population bottleneck. In this regard, our results show low genetic diversity in both species with a single predominant cluster of *P. falciparum* and sub-structured *P. vivax* populations in the peri-urban settings of Iquitos (Loreto, Peru). PMC control activities in Peru had a higher impact against the *P. falciparum* population than *P. vivax* as previously shown in other regions^50^. This difference could be due to the lower prevalence and absence of hypnozoites in *P. falciparum*^51,52^. In addition, the lower treatment adherence for *P. vivax* in Peru^53,54^ may have also played a role in the differential scenarios for both species^51,52^.

Clinical and epidemiological data derived from our study showed no significant differences between participants carrying *P. vivax* and *P. falciparum* parasites. In this regard, most subjects were male with the most common symptoms being a headache, malaise, chills, fever and sweating which were present in more than 85% of all subjects. This lack of severity in *P. falciparum* and *P. vivax* is characteristic in the region compared to Africa or Southeast Asia where *P. falciparum* is associated with more severe symptoms^55^.

Previous studies have shown that antifolate drugs have exerted strong selective pressures on *P. vivax*^56,57^. In this regard, our results show a high proportion of putative SP resistance markers 58R and 117N on *Pvdhfr* and 383G *Pvdhps*^25–27^. This high proportion could result from indirect selection pressure from *P. falciparum* treatment. Noteworty, SP was removed as first line treatment against *P. falciparum* in the study region in 2001^58^. Therefore, *Pvdhfr* and *Pvdhps* mutations remain fixed for more than 20 years. In the case of *Pvmdr1*, there is a low prevalence of the putative CQ resistant marker 976F in the *P. vivax* population (4.7%). However, it is important to notice that the role of the Y976F on CQ resistance remains under debate^27,59,60^.

Currently, there is concern regarding the spread of artemisinin resistance which could halter malaria elimination efforts around the globe^61^. Our study did not find evidence of artemisinin resistance nor related mutations on *P. falciparum* in Iquitos. This result and the lack of reports of clinical resistance nor delayed parasite clearance indicate that artemisinin remains effective in the region. Although continuing surveillance and increased sample sizes are needed to detect early resistance signals.

Regarding other drug resistance markers and haplotypes, our results show that most *P. falciparum* parasites presented a profile of drug resistance mutations compatible with the foreign PfBv1 lineage. This profile includes the CQ resistance 184F/1246Y double mutation on *Pfmdr1*^62^ and the SVMNT haplotype on *Pfcrt*^34^ and the SP resistant quadruple mutation 51I/108N + 437G/540E at *Pfdhfr* and *Pfdhps*^32^. This lineage was found on a *P. falciparum* outbreak in the North Coast of Peru between 2010 and 2012^12^ and in another outbreak in the region of Cusco in 2013^11^. In this regard, our population structure results for *P. falciparum* indicate that up to 95% of our samples belong to PfBv1 which appears to have become the predominant lineage in the study region.

Our results show that most samples were monoclonal and there was a low intrapopulation diversity (π) for both species. However, *P. falciparum* had a much lower value than *P. vivax* which indicates that circulating *P. falciparum* are less admixed and predominantly clonal. This result is consistent with the population structure results for both species which did not show clustering according to geographical locations and the presence of a single *P. falciparum* cluster that accounted for 95% of all infections. The differential collection types of the samples plus the restricted number of samples could mask sub-structuring due to geographical origin. In the case of *P. vivax*, our results reflect the higher diversity with three different circulating genetic clusters, which share the same habitats and 21 admixed individuals. This could reflect a scenario with more gene flow between the current Amazonian communities compared to results from previous years probably due to higher connectivity between communities^15,16^. Furthermore, the haplotype network for *P. vivax* showed a closer relationship with some Colombian samples. This finding points towards genetic exchange across these countries derived from human migration and high connectivity across the Peruvian/Colombian borders.

Malaria elimination programs face the challenge of drug resistance^7^ and introduction of new parasite lineages^63^. In this regard, the *P. falciparum* predominant cluster (Bv1) could have been introduced from Brazil or Bolivia where the genotypes *Pfdhr* 50R, 51I and 108N and *Pfcrt* 72S, 74M, 75N, 76T, 326D and 356L were initially described^9,34,63^. There is evidence that parasites harboring this haplotype have displaced local populations and caused outbreak in different endemic regions in South America^10–12,63^.

In *P. vivax*, the three most frequent haplotypes represented more than 59% of all our samples and the prevalence of these populations was associated with *Pvdhfr* and *Pvdhps* mutations*.* In this regard, the presence of these mutations could have provided a selective advantage over other circulating populations during the period of SP use for *P. falciparum* and mixed infections in the Peruvian Amazon (1996–2001) and in the Peruvian North Coast (1996–2015)^58,64^.

Unfortunately, analyses of *P. vivax* in the context of our study are limited due to the short barcode for this species (38 SNPs). Also, our study was restricted to a brief period of time and therefore we cannot assess the dynamics of circulating parasite populations over time. Finally, our study was executed prior the COVID 19 pandemic and it is highly likely that disruptions derived from the pandemic could have impacted the dynamics of malaria transmission in the region.

In summary, our study shows a low genetic diversity for both species, sub-structured *P. vivax* populations, clonal propagation in *P. falciparum* and absence of artemisinin resistance mutations in the peri-urban settings of Iquitos. The lack of *P. vivax* sub-structuring due to geographic origin reflects a higher rate of gene flow among the geographic units compared to previous years. Therefore, the new malaria elimination plan must consider developing strategies that address human mobility as a high risk for malaria transmission and persistence. Moreover, our findings support the continuity of ACTs in the region and highlight the need to adapt the elimination strategies to decreased *P. vivax* incidence.

## Supplementary Information

Supplementary Information.

## Data Availability

The dataset generated during and/or analyzed during the current study is available from the corresponding author on reasonable request. Raw sequence data has been deposited at the European Nucleotide Archive (https://www.ebi.ac.uk/ena/browser/home) under primary accession numbers from ERS2741604 to ERS3516726.
