# Supplementary material for: Drug resistance and population structure of Plasmodium falciparum and Plasmodium vivax in the Peruvian Amazon
Source: Sci Rep. 2022 Oct 1;12:16474. doi: 10.1038/s41598-022-21028-3 (PMC9526214; doi:10.1038/s41598-022-21028-3)
Supplement: Supplementary file 1 — Supplementary Information. [file 41598_2022_21028_MOESM1_ESM.pdf]

Supplementary files

Drug resistance and population structure of Plasmodium falciparum and Plasmodium vivax in the Peruvian Amazon

Fredy E. Villena, Juan F. Sanchez, Oscar Nolasco, Greys Braga, Leonila Ricopa, Keare Barazorda, Carola J. Salas, Carmen Lucas, Stephen E. Lizewski, Christie A. Joya, Dionicia Gamboa, Christopher Delgado-Ratto, Hugo O. Valdivia

S1 figure: Median-joining network for P. vivax samples from Peru (blue), Colombia (green) and Honduras (purple). The sizes of the circles are proportional to the number of haplotype sequences. The number of straight lines connecting the haplotypes is proportional to the number of mutational steps. Figure generated in PopArt (http://popart.otago.ac.nz/index.shtml).

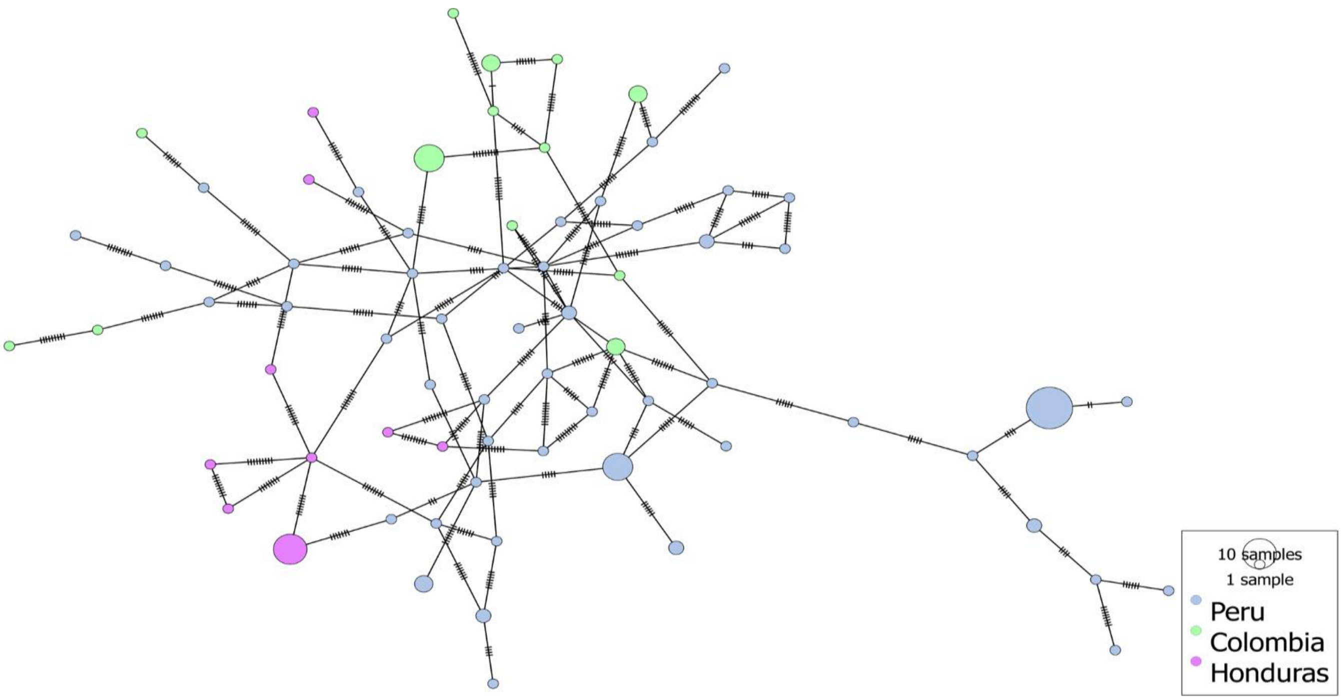

S1 Table: Drug resistant mutations on *P. falciparum*. The table aminoacid codes for multiple drug resistance associated genes on *P. falciparum*. Colors on the amino acids indicate amino acid change according to the Zappo color scheme (Aliphatic/hydrophobic in pink, aromatic in orange, positive in blue, negative in red, hydrophilic in green, conformational special in magenta and cysteine in yellow).

N°	Code	Kelch13	DHFR				DHPS					CRT							EXO	MDR1			ARP-S10		FERREDOXIN	MDR2
		BTB/POZ and propeller	N 51	C 59	S 108	I 164	S 436	A 437	K 540	A 581	A 613	C 72	V 73	M 74	N 75	K 76	N 326	I 356	E 415	N 86	Y 184	D 1246	V 127	D 128	D 193	T 484
1	MDP6485	-	I	C	N	I	S	G	E	G	A	S	V	M	N	T	D	L	E	N	F	Y	V	D	D	T
2	MDP6467	WT	I	C	N	I	S	G	E	G	A	S	V	M	N	T	D	L	E	N	F	Y	V	D	D	T
3	MDP6435	WT	I	C	N	I	S	G	E	G	A	S	V	M	N	T	D	L	E	N	F	Y	V	D	D	T
4	MDP6430	WT	I	C	N	I	S	G	E	G	A	S	V	M	N	T	D	L	E	N	F	Y	V	D	D	T
5	MDP6429	-	I	C	N	I	-	-	-	-	-	S	V	M	N	T	D	L	E	N	F	Y	V	D	D	T
6	MDP6424	-	I	C	N	I	S	G	[E/N]	[A/G]	A	S	V	M	N	T	D	L	E	N	F	[D/Y]	V	D	D	T
7	MDP6422	-	I	C	N	I	S	G	E	G	A	S	V	M	N	T	D	L	E	N	F	Y	V	D	D	T
8	MDP6400	WT	I	-	N	I	S	G	-	G	A	S	V	M	N	T	D	L	E	-	-	-	V	D	D	T
9	MDP6398	-	I	C	N	I	[S/A]	G	E	G	A	S	V	M	N	T	D	L	E	N	F	Y	V	D	D	T
10	MDP6391	-	I	C	N	I	S	G	E	G	A	S	V	M	N	T	D	L	E	N	F	Y	V	D	D	T
11	MDP6389	-	I	C	N	I	S	G	E	G	A	S	V	M	N	T	D	L	E	N	F	Y	V	D	D	T
12	MDP6381	WT	I	C	N	I	S	G	E	G	A	S	V	M	N	T	D	L	E	-	F	Y	V	D	D	T
13	MDP6378	WT	I	C	N	I	S	G	E	G	A	S	V	M	N	T	D	L	E	N	F	Y	V	D	D	T
14	MDP6377	-	I	C	N	I	S	G	E	G	A	S	V	M	N	T	D	L	E	-	F	Y	V	D	D	T
15	MDP6376	WT	I	C	N	I	S	G	E	G	A	S	V	M	N	T	D	L	E	N	F	Y	V	D	D	T
16	MDP6374	WT	I	C	N	I	S	G	E	G	A	S	V	M	N	T	D	L	E	N	-	-	V	D	D	T
17	MDP6362	WT	I	C	N	I	S	G	[K/E]	G	A	S	V	M	N	T	D	L	E	N	F	Y	V	D	D	T
18	MDP6346	WT	I	C	N	I	S	G	E	G	A	S	V	M	N	T	D	L	E	N	F	Y	V	D	D	T
19	MDP6342	WT	I	C	N	I	S	G	E	G	A	S	V	M	N	T	D	L	E	N	F	Y	V	D	D	T
20	MDP6327	WT	I	C	N	I	S	G	E	G	A	S	V	M	N	T	D	L	E	N	F	Y	V	D	D	T
21	MDP6322	-	I	C	N	I	S	G	-	-	-	S	V	M	N	T	D	L	E	N	-	Y	V	D	D	T
22	MDP6317	-	I	C	-	-	S	G	E	G	A	S	V	M	N	T	D	L	E	N	-	Y	V	D	D	T
23	MDP6316	-	I	C	N	I	S	G	-	-	-	S	V	M	N	T	D	L	E	N	F	Y	V	D	D	T
24	MDP6306	-	I	C	N	I	S	G	E	G	A	S	V	M	N	T	D	L	E	N	F	[D/Y]	V	D	D	T
25	MDP6305	WT	I	C	N	I	S	G	E	G	A	S	V	M	N	T	D	L	E	N	F	Y	V	D	D	-
26	MDP6299	WT	I	C	N	I	S	G	[K/E]	-	A	S	V	M	N	T	D	L	E	N	F	Y	V	D	D	T
27	MDP6296	WT	I	C	N	I	S	G	E	G	A	S	V	M	N	T	D	L	E	N	F	Y	V	D	D	T
28	MDP6293	WT	I	C	N	I	S	G	E	G	A	S	V	M	N	T	D	L	E	N	F	Y	V	D	D	T
29	MDP6292	WT	I	C	N	I	S	G	E	G	A	S	V	M	N	T	D	L	E	N	F	Y	V	D	D	T
30	MDP6290	-	I	C	N	I	S	G	[K/E]	G	A	S	V	M	N	T	D	L	E	N	F	Y	V	D	D	T
31	MDP6289	WT	I	C	N	I	S	G	E	G	A	S	V	M	N	T	D	L	E	N	F	Y	V	D	D	T
32	MDP6287	WT	-	-	-	-	[S/A]	G	-	G	A	S	V	M	N	T	D	L	E	N	F	-	V	D	D	-
33	MDP6285	WT	I	C	N	I	-	G	[E/N]	-	-	S	V	M	N	T	D	L	E	N	F	-	V	D	D	T
34	MDP6283	-	I	C	N	I	-	-	-	-	-	S	V	M	N	T	D	L	E	N	-	[D/Y]	V	D	D	T
35	MDP6281	WT	I	C	N	I	S	G	E	G	A	S	V	M	N	T	D	L	E	N	F	Y	V	D	D	T
36	MDP6271	WT	I	C	N	I	S	G	E	G	A	S	V	M	N	T	D	L	E	N	F	Y	V	D	D	T
37	MDP6257	WT	I	C	N	I	S	G	E	G	A	S	V	M	N	T	D	L	E	N	F	Y	V	D	D	T
38	MDP4996	WT	I	C	N	I	S	G	-	G	A	S	V	M	N	T	D	L	E	-	F	-	V	D	D	T
39	MDP4989	A617S	I	C	N	I	S	G	E	G	A	S	V	M	N	T	D	L	E	-	-	-	V	D	D	T
40	MDP4987	WT	I	C	N	I	S	G	E	G	A	S	V	M	N	T	D	L	E	N	F	Y	V	D	D	T
41	MDP4986	WT	I	C	N	I	S	G	E	G	A	S	V	M	N	T	D	L	E	N	F	Y	V	D	D	T
42	MDP4983	-	[N/I]	C	N	I	S	G	E	G	A	[C/S]	V	M	N	T	D	L	E	N	-	Y	V	D	D	T
43	MDP4974	WT	I	C	N	I	S	G	E	G	A	[C/S]	V	M	N	T	D	L	E	N	F	[D/Y]	V	D	D	T
44	MDP4970	WT	I	C	N	I	S	G	E	G	A	S	V	M	N	T	D	L	E	N	F	[D/Y]	V	D	D	T
45	MDP4950	WT	I	C	N	I	S	G	E	-	-	S	V	M	N	T	D	L	E	N	F	Y	V	D	D	T
46	MDP4937	WT	I	C	N	I	S	G	E	G	A	S	V	M	N	T	D	L	E	N	F	Y	V	D	D	T
47	MDP4921	-	I	C	N	I	S	G	E	G	A	S	V	M	N	T	D	L	E	N	F	Y	V	D	D	T
48	MDP4918	-	I	C	N	I	S	G	E	G	A	S	V	M	N	T	D	L	E	N	F	Y	V	D	D	T
49	MDP4917	-	I	C	N	I	S	G	E	G	A	S	V	M	N	T	D	L	E	N	F	Y	V	D	D	T
50	MDP4916	WT	I	C	N	I	S	G	E	G	A	S	V	M	N	T	D	L	E	N	F	Y	V	D	D	T
51	MDP4546	WT	I	C	N	I	S	-	E	G	A	S	V	M	N	T	D	L	E	N	F	Y	V	D	D	T
52	MDP4393	WT	I	C	N	I	S	A	K	A	A	C	V	M	N	T	D	L	E	N	F	[D/Y]	V	D	D	T
53	MDP3907	WT	I	C	N	I	S	G	E	G	A	S	V	M	N	T	D	L	E	N	F	Y	V	D	D	T
54	9072051	-	I	C	N	I	S	G	E	G	A	S	V	M	N	T	D	L	E	N	F	Y	V	D	D	T
55	9071461	WT	I	C	N	I	S	G	E	G	A	S	V	M	N	T	D	L	E	N	F	Y	V	D	D	T
56	9071451	WT	I	C	N	I	S	G	E	G	A	S	V	M	N	T	D	L	E	N	F	Y	V	D	D	T
57	9061131	WT	I	C	N	I	S	G	E	G	A	S	V	M	N	T	D	L	E	N	F	Y	V	D	D	T
58	9050451	WT	I	C	N	I	S	G	E	G	A	S	V	M	N	T	D	L	E	N	F	Y	V	D	D	T
59	503290	WT	I	C	N	I	S	G	E	G	A	S	V	M	N	T	D	L	E	N	F	Y	V	D	D	T
60	503259	WT	I	C	N	I	S	G	E	G	A	S	V	M	N	T	D	L	E	N	F	Y	V	D	D	T
61	503257	WT	I	C	N	I	S	G	E	G	A	S	V	M	N	T	D	L	E	N	F	Y	V	D	D	T
62	503253	WT	I	C	N	I	S	G	E	-	-	S	V	M	N	T	D	L	E	N	-	-	V	D	D	T
63	503234	WT	I	C	N	I	S	G	E	G	A	S	V	M	N	T	D	L	E	N	F	Y	V	D	D	T

S2 Table: Drug resistant haplotypes on *P. falciparum*. The table shows the frequency of haplotypes on drug resistance genes PfMDR1, PfDHFR, PfDHPS, PfCRT, PfARPS-10, PfEXO, FERREDOXIN, PfMDR2, Pfk13.

Gene	Position	Haplotype	n (%)
PfMDR1	86/184/1246	N**FY**	45 (71.4)
		N**F**[D/**Y**]	5 (7.9)
		N**F**-	2 (3.2)
		N-**Y**	3 (4.8)
		-**FY**	2 (3.2)
		-**F**-	1 (1.6)
		N- -	2 (3.2)
		N-[D/**Y**]	1 (1.6)
		- - -	2 (3.2)
PfDHFR	51/59/108/164	**I**C**N**I	59 (93.6)
		**I**C- -	1 (1.6)
		**I**-**N**I	1 (1.6)
		**[N/I]CNI**	1 (1.6)
		- - - -	1 (1.6)
PfDHPS	436/437/540/581/613	S**GEG**A	46 (73)
		S**G**-**G**A	2 (3.2)
		S**G**- - -	2 (3.2)
		SAKAA	1 (1.6)
		S**G[E/N]**[A/**G**]A	1 (1.6)
		S**G**[K/**E**]**G**A	2 (3.2)
		[S/**A**]**GEG**A	1 (1.6)
		S**G**[K/**E**]-A	1 (1.6)
		[S**/A**]**G**-**G**A	1 (1.6)
		S-EGA	1 (1.6)
		S**GE**- -	2 (3.2)
		-**G[E/N]**- -	1 (1.6)
		- - - - -	2 (3.2)
PfCRT	72/73/74/75/76/326/356	**S**VMN**TDL**	60 (95.2)
		CVMN**TDL**	1 (1.6)
		[C/**S**]VMN**TDL**	2 (3.2)
PfARPS-10	127/128	VD	63 (100)
PfEXO	415	E	63 (100)
FERREDOXIN	193	D	63 (100)
PfMDR2	484	T	61 (96.8)
		-	2 (3.1)
Pfk13	BTB/POZ and propeller	WT	43 (68.3)
		**A617S**	1 (1.6)
		-	19 (30.1)
PfDHFR/DHPS	*PfDHFR* 51/59/108/164<br>*PfDHPS* 436/437/540/581/613	**I**C**N**IS**GEG**A	44 (69.8)
		**I**C**N**IS**G**[K/**E**]**G**A	2 (3.2)
		**I**C**N**ISAKAA	1 (1.6)
		**I**C**N**IS**G**[**E**/N][A/**G**]A	1 (1.6)
		**I**C**N**I[S/**A**]**GEG**A	1 (1.6)
		[**N**/I]C**N**IS**GEG**A	1 (1.6)
		**I**C**N**IS-**EG**A	1 (1.6)
		**I**C**N**IS**G**-**G**A	1 (1.6)
		**I**C**N**IS**G**[K/**E**]-A	1 (1.6)
		**I**-**N**IS**G**-**G**A	1 (1.6)
		**I**C--S**GEG**A	1 (1.6)
		**I**C**N**IS**GE**--	2 (3.2)
		**I**C**N**IS**G**---	2 (3.2)
		**I**C**N**I-**G**[**E**/N]--	1 (1.6)
		**I**C**N**I-----	2 (3.2)
		----[S/**A**]**G**-**G**A	1 (1.6)

PfMDR1/CRT	*PfMDR1* 86/184/1246 *PfCRT* 72/73/74/75/76/326/356	N**FYS**VMN**TDL**	45 (71.4)
		N**F**[D/**Y**]**S**VMN**TDL**	3 (4.8)
		N**F**[D/**Y**][C/**S**]VMN**TDL**	1 (1.6)
		N**F**[D/**Y**]CVMN**TDL**	1 (1.6)
		N-**YS**VMN**TDL**	2 (3.2)
		N**F**-**S**VMN**TDL**	2 (3.2)
		-**FYS**VMN**TDL**	2 (3.2)
		N-[D/**Y**]**S**VMN**TDL**	1 (1.6)
		N-**Y**[C/**S**]VMN**TDL**	1 (1.6)
		N--**S**VMN**TDL**	2 (3.2)
		-**F**-**S**VMN**TDL**	1 (1.6)
		---**S**VMN**TDL**	2 (3.2)
PfDHFR/DHPS/CRT/EXO/ MDR1/ARPS-10/FERREDOXIN/MDR2	*PfDFFR* 51/59/108/164 *PfDHPS* 436/437/540/581/613 CRT 72/73/74/75/76/326/356 *PfEXO* 415 *PfMDR1* 86/184/1246 *PfARPS-10* 127/128 *FERREDOXIN* 193 *PfMDR2* 484	**I**C**N**IS**GEG**A**S**VMN**TDL**EN**FY**VDDT	36 (57.1)
		**I**C**N**IS**G**[K/**E**]**G**A**S**VMN**TDL**EN**FY**VDDT	2 (3.2)
		**I**C**N**IS**GEG**A**S**VMN**TDL**EN**F**[D/**Y**]VDDT	2 (3.2)
		**I**C**N**IS**GEG**A**S**VMN**TDL**E-**FY**VDDT	2 (3.2)
		**I**C**N**I[S/**A**]**GEG**A**S**VMN**TDL**EN**FY**VDDT	1 (1.6)
		**I**C**N**ISAKAACVMN**TDL**EN**F**[D/**Y**]VDDT	1 (1.6)
		**I**C**N**IS**GEG**A[C/**S**]VMN**TDL**EN**F**[D/**Y**]VDDT	1 (1.6)
		**I**C**N**IS**G**[**E**/N][A/**G**]A**S**VMN**TDL**EN**F**[D/**Y**]VDDT	1 (1.6)
		**I**C**N**IS-**EG**A**S**VMN**TDL**EN**FY**VDDT	1 (1.6)
		[N/**I**]C**N**IS**GEG**A[C/**S**]VMN**TDL**EN-**Y**VDDT	1 (1.6)
		**I**C**N**IS**G**[K/**E**]-A**S**VMN**TDL**EN**FY**VDDT	1 (1.6)
		**I**C**N**IS**GEG**A**S**VMN**TDL**EN**FY**VDD-	1 (1.6)
		**I**C--S**GEG**A**S**VMN**TDL**EN-**Y**VDDT	1 (1.6)
		**I**C**N**IS**G**-**G**A**S**VMN**TDL**E-**F**-VDDT	1 (1.6)
		**I**C**N**IS**G**---**S**VMN**TDL**EN**FY**VDDT	1 (1.6)
		**I**C**N**IS**GE**--**S**VMN**TDL**EN**FY**VDDT	1 (1.6)
		**I**C**N**IS**GEG**A**S**VMN**TDL**E---VDDT	1 (1.6)
		**I**C**N**IS**GEG**A**S**VMN**TDL**EN--VDDT	1 (1.6)
		**I**-**N**IS**G**-**G**A**S**VMN**TDL**E---VDDT	1 (1.6)
		**I**C**N**I-**G**[**E**/N]--**S**VMN**TDL**EN**F**-VDDT	1 (1.6)
		**I**C**N**I-----**S**VMN**TDL**EN-[D/**Y**]VDDT	1 (1.6)
		**I**C**N**IS**G**---**S**VMN**TDL**EN-**Y**VDDT	1 (1.6)
		**I**C**N**IS**GE**--**S**VMN**TDL**EN--VDDT	1 (1.6)
		**I**C**N**I-----**S**VMN**TDL**EN**FY**VDDT	1 (1.6)
		----[S/A]**G**-**G**A**S**VMN**TDL**EN**F**-VDD-	1 (1.6)

S3 Table: Drug resistant mutations on *P. vivax*. The table shows individual aminoacids on *Pvdhfr, Pvdhps* and *Pvmdr1*. Colors on the amino acids indicate amino acid change according to the Zappo color scheme (Aliphatic/hydrophobic in pink, aromatic in orange, positive in blue, negative in red, hydrophilic in green, conformational special in magenta and cysteine in yellow).

N°	Code	PvDHFR				PvDHPS					PvMDR1
		F 57	S 58	T 61	S 117	E 380	S 382	A 383	Y 385	A 553	Y 976
1	501118	F	R	T	N	E	C	G	Y	A	Y
2	502231	F	R	T	N	E	C	G	Y	A	Y
3	503054	F	R	T	N	E	C	G	Y	A	Y
4	503075	F	K	T	N	E	C	G	Y	A	Y
5	503088	F	K	T	N	E	C	G	Y	A	Y
6	503119	F	K	T	N	E	S	G	Y	A	Y
7	503121	F	K	T	N	E	C	G	Y	A	Y
8	503219	F	R	T	N	E	C	G	Y	A	Y
9	503239	F	K	T	N	E	C	G	Y	A	Y
10	MDP6162	F	[R/S]	T	N	E	S	A	Y	A	Y
11	503250	F	K	T	N	E	C	G	Y	A	Y
12	503260	F	R	T	N	E	S	A	Y	A	Y
13	503280	F	K	T	N	E	C	G	Y	A	Y
14	503285	F	K	T	N	E	C	G	Y	A	Y
15	503287	F	R	T	N	E	C	G	Y	A	Y
16	MDP4394	F	R	T	N	E	S	A	Y	A	Y
17	MDP4506	F	R	T	N	E	S	G	Y	A	Y
18	MDP4554	F	R	T	N	E	S	A	Y	A	Y
19	MDP4556	F	R	T	N	E	S	[G/A]	Y	A	Y
20	MDP4864	F	R	T	N	E	C	G	Y	A	Y
21	MDP4865	F	S	T	N	E	S	A	Y	A	Y
22	MDP4869	F	K	T	N	E	S	A	Y	A	Y
23	MDP4881	F	R	T	N	E	S	G	Y	A	Y
24	MDP4882	F	K	T	N	E	C	G	Y	A	Y
25	MDP4914	F	K	T	N	E	S	G	Y	A	Y
26	MDP4927	F	K	T	N	E	S	A	Y	A	Y
27	MDP4929	F	R	T	N	E	S	G	Y	A	Y
28	MDP4930	F	R	T	N	E	S	A	Y	A	Y
29	MDP4931	F	K	T	N	E	S	A	Y	A	Y
30	MDP4936	F	R	T	N	E	S	A	Y	A	F
31	MDP4938	F	K	T	N	E	S	A	Y	A	Y
32	MDP4940	F	R	T	N	E	S	[G/A]	Y	A	Y
33	MDP4952	F	R	T	N	E	C	G	Y	A	F
34	MDP4954	F	K	T	N	E	C	G	Y	A	Y
35	MDP4961	F	R	T	N	E	C	G	Y	A	Y
36	MDP4968	F	R	T	N	E	C	G	Y	A	Y
37	MDP4978	F	R	T	N	E	S	A	Y	A	Y
38	MDP4982	F	K	T	N	E	S	A	Y	A	Y
39	MDP4985	F	R	T	N	E	C	G	Y	A	Y
40	MDP4988	F	[R/L]	T	N	E	S	G	Y	A	Y
41	MDP4990	F	R	T	N	E	C	G	Y	A	Y
42	MDP4991	F	K	T	N	E	S	G	Y	A	Y
43	MDP4992	F	K	T	N	E	C	G	Y	A	Y
44	MDP6160	F	R	T	N	E	C	G	Y	A	Y
45	MDP6204	F	S	T	S	E	S	A	Y	A	Y
46	MDP6208	F	K	T	N	E	C	G	Y	A	Y
47	MDP6212	F	R	T	N	E	S	A	Y	A	F
48	MDP6215	F	S	T	N	E	S	A	Y	A	Y
49	MDP6232	F	R	T	N	E	S	A	Y	A	Y
50	MDP6233	F	R	T	N	E	C	G	Y	A	Y
51	MDP6240	F	K	T	N	E	C	G	Y	A	Y
52	MDP6241	F	R	T	N	E	S	G	Y	A	Y
53	MDP6245	F	K	T	N	E	C	G	Y	A	Y
54	MDP6249	F	K	T	N	E	C	G	Y	A	Y
55	MDP6261	F	R	T	N	E	C	G	Y	A	Y

56	MDP6262	F	K	T	N	E	C	G	Y	A	Y
57	MDP6273	F	K	T	N	E	S	A	Y	A	Y
58	MDP6277	F	K	T	N	E	C	G	Y	A	Y
59	MDP6279	F	R	T	N	E	S	G	Y	A	Y
60	MDP6284	F	R	T	N	E	C	G	Y	A	F
61	MDP6286	F	K	T	N	E	C	G	Y	A	Y
62	MDP6291	F	K	T	N	E	S	G	Y	A	Y
63	MDP6294	F		T	N	E	S	A	Y	A	Y
64	MDP6297	F	R	T	N	E	C	G	Y	A	Y
65	MDP6300	F	K	T	N	E	S	A	Y	A	Y
66	MDP6301	F	K	T	N	E	C	G	Y	A	Y
67	MDP6302	F	R	T	N	E	S	G	Y	A	Y
68	MDP6308	F	R	T	N	E	S	A	Y	A	Y
69	MDP6310	F	K	T	N	E	C	G	Y	A	Y
70	MDP6313	F	K	T	N	E	C	G	Y	A	Y
71	MDP6314	F	R	T	N	E	S	A	Y	A	Y
72	MDP6323	F	R	T	N	E	S	A	Y	A	Y
73	MDP6324	F	K	T	N	E	C	G	Y	A	Y
74	MDP6325	F	R	T	N	E	C	G	Y	A	Y
75	MDP6326	F	K	T	N	E	C	G	Y	A	Y
76	MDP6328	F	R	T	N	E	S	A	Y	A	Y
77	MDP6329	F	R	T	N	E	C	G	Y	A	Y
78	MDP6330				N	E	C	G	Y	A	Y
79	MDP6347				N	E	[S/C]	[G/A]	Y	A	Y
80	MDP6331	F	R	T	N	E	S	G	Y	A	Y
81	MDP6332	F	R	T	N	E	C	G	Y	A	F
82	MDP6336	F	K	T	N	E	C	G	Y	A	Y
83	MDP6385				N	E	C	G	Y	A	Y
84	MDP6338	F	R	T	N	E	S	A	Y	A	Y
85	MDP6339	F	R	T	N	E	S	G	Y	A	Y
86	MDP6351	F		T	N	E	[S/C]	[G/A]	Y	A	Y
87	MDP6340	F		T	N	E	S	G	Y	A	Y
88	MDP6341				N	E	S	G	Y	A	Y
89	MDP6343	F	K	T	N	E	C	G	Y	A	Y
90	MDP6344	F	K	T	N	E	C	G	Y	A	Y
91	MDP6349	F	R	T	N	E	S	G	Y	A	Y
92	MDP6350	F	K	T	N	E	C	G	Y	A	Y
93	MDP6353	F	K	T	N	E	S	A	Y	A	Y
94	MDP6354				S	E	S	A	Y	A	Y
95	MDP6360	F	[R/L]	T	N	E	[S/C]	G	Y	A	[F/Y]
96	MDP6355				S	E	S	A	Y	A	Y
97	MDP6356	L	R	T	S	E	S	A	Y	A	Y
98	MDP6357	F	R	T	N	E	C	G	Y	A	Y
99	MDP6358	F	K	T	N	E	C	G	Y	A	Y
100	MDP6359	F	K	T	N	E	C	G	Y	A	Y
101	MDP6361	F	R	T	N	E	C	G	Y	A	Y
102	MDP6364	F	K	T	N	E	C	G	Y	A	Y
103	MDP6366				N	E	S	A	Y	A	Y
104	MDP6369	F	K	T	N	E	C	G	Y	A	Y
105	MDP6370	F	K	T	N	E	C	G	Y	A	Y
106	MDP6372	F	R	T	N	E	C	G	Y	A	Y
107	MDP6375	F	K	T	N	E	C	G	Y	A	Y
108	MDP6383	F	K	T	N	E	C	G	Y	A	Y
109	MDP6384	F	K	T	N	E	S	A	Y	A	Y
110	MDP6390	F	K	T	N	E	S	A	Y	A	Y
111	MDP6426	F	R	T	N	E	S	A	Y	A	Y
112	MDP6393	F	K	T	N	E	C	G	Y	A	Y
113	MDP6395	F	K	T	N	E	S	G	Y	A	Y
114	MDP6396	F	R	T	N	E	C	G	Y	A	Y
115	MDP6397	F	[R/L]	T	N	E	C	G	Y	A	Y
116	MDP6399	F	R	T	N	E	C	G	Y	A	Y

117	MDP6441	F	K	T	N	E	[S/C]	G	Y	A	Y
118	MDP6401	F	K	T	N	E	C	G	Y	A	Y
119	MDP6402	F	K	T	N	E	C	G	Y	A	Y
120	MDP6403	F	K	T	N	E	C	G	Y	A	Y
121	MDP6405	F	K	T	N	E	S	A	Y	A	Y
122	MDP6407	F	R	T	N	E	S	A	Y	A	Y
123	MDP6408	F	K	T	N	E	C	G	Y	A	Y
124	MDP6410	F	K	T	N	E	C	G	Y	A	Y
125	MDP6411	F	K	T	N	E	C	G	Y	A	Y
126	MDP6412	F	K	T	N	E	C	G	Y	A	Y
127	MDP6415	F	R	T	N	E	C	G	Y	A	Y
128	MDP6416	F	[R/K]	T	N	E	C	G	Y	A	Y
129	MDP6420	F	K	T	N	E	C	G	Y	A	Y
130	MDP6421	F	R	T	N	E	C	G	Y	A	Y
131	MDP6427				N	E	S	A	Y	A	Y
132	MDP6431	F	K	T	N	E	S	A	Y	A	Y
133	MDP6432	F	R	T	N	E	C	G	Y	A	Y
134	MDP6434	F	R	T	N	E	C	G	Y	A	F
135	MDP6436	F	K	T	N	E	S	G	Y	A	Y
136	MDP6440	F	R	T	N	E	S	A	Y	A	Y
137	MDP6442				N	E	C	G	Y	A	Y
138	MDP6447	F	K	T	N	E	C	G	Y	A	Y
139	MDP6448	F	R	T	N	E	S	G	Y	A	Y
140	MDP6449	F	K	T	N	E	C	G	Y	A	Y
141	MDP6451	F	K	T	N	E	S	G	Y	A	Y
142	MDP6452	F	K	T	N	E	C	G	Y	A	Y
143	MDP6455	F	R	T	N	E	S	A	Y	A	Y
144	MDP6460	F	R	T	N	E	S	G	Y	A	Y
145	MDP6462	F	R	T	N	E	C	G	Y	A	Y
146	MDP6469	F	K	T	N	E	C	G	Y	A	Y
147	MDP6471	F	K	T	N	E	S	A	Y	A	Y
148	MDP6480	F	R	T	N	E	S	A	Y	A	Y
149	MDP6481	F	R	T	N	E	C	G	Y	A	Y
150	MDP6486	F	K	T	N	E	C	G	Y	A	Y
151	MDP6522	F	[R/L]	T	N	E	[S/C]	[G/A]	Y	A	Y
152	MDP6491	F	R	T	N	E	S	A	Y	A	Y
153	MDP6492	F	R	T	N	E	S	G	Y	A	Y
154	MDP6496	F	K	T	N	E	C	G	Y	A	Y
155	MDP6565	F	K	T	N	E	[S/C]	[G/A]	Y	A	Y
156	MDP6498	F	R	T	N	E	S	G	Y	A	Y
157	MDP6499	F	R	T	N	E	S	G	Y	A	Y
158	MDP6500	F	S	T	S	E	S	A	Y	A	Y
159	503073	F	K	T	N	E	[S/C]	[G/A]	Y	A	Y
160	MDP6501	F	R	T	N	E	C	G	Y	A	Y
161	MDP6502	F	K	T	N	E	C	G	Y	A	Y
162	MDP6504	F	K	T	N	E	C	G	Y	A	Y
163	MDP6511	F	R	T	N	E	S	A	Y	A	Y
164	MDP6512	F	R	T	N	E	S	A	Y	A	Y
165	MDP6513	F	K	T	N	E	C	G	Y	A	Y
166	MDP6517	F	K	T	N	E	S	G	Y	A	Y
167	MDP6520	F	R	T	N	E	S	A	Y	A	Y
168	MDP6556	F	K	T	N	E	C	G	Y	A	Y
169	MDP6564	F	K	T	N	E	C	G	Y	A	Y
170	MDP6566	F	R	T	N	E	S	A	Y	A	Y

S4 Table: Putative drug resistant haplotypes on *P. vivax*. The table shows the frequency of haplotypes on putative drug resistance genes *PvDHFR, PvDHPS, PvMDR1.*

Gene	Position	Haplotype	n (%)
PvMDR1	976	Y	163 (95.9)
		**F**	6 (3.5)
		[**F**/Y]	1 (0.6)
PvDHFR	57/58/61/117	F**K**T**N**	77 (45.3)
		F**R**T**N**	70 (41.2)
		F[**R/L**]T**N**	4 (2.4)
		F-T**N**	3 (1.8)
		FST**N**	2 (1.2)
		FSTS	2 (1.2)
		**LR**TS	1 (0.59)
		F[**R/K**]T**N**	1 (0.59)
		F[**R**/S]T**N**	1 (0.59)
		- - -S	2 (1.2)
		- - -**N**	7 (4.1)
PvDHPS	380/382/383/385/553	E**CG**YA	89 (52.4)
		ESAYA	47 (27.6)
		ES**G**YA	25 (14.7)
		E[S/**C**][**G**/A]YA	5 (2.94)
		E[S/**C**]**G**YA	2 (1.2)
		ES[**G**/A]YA	2 (1.2)
PvDHFR/DHPS	DHFR 57/58/61/117 DHPS 380/382/383/385/553	F**K**T**N**E**CG**YA	53 (31.2)
		F**R**T**N**E**CG**YA	31 (18.2)
		F**R**T**N**ESAYA	23 (13.5)
		F**R**T**N**ES**G**YA	14 (8.2)
		F**K**T**N**ESAYA	13 (7.6)
		F**K**T**N**ES**G**YA	8 (4.7)
		- - -**N**E**CG**YA	3 (1.77)
		F**R**T**N**ES**[G**/A]YA	2 (1.2)
		FST**N**ESAYA	2 (1.2)
		FSTSESAYA	2 (1.2)
		- - -**N**ESAYA	2 (1.2)
		- - -SESAYA	2 (1.2)
		F[**R/K**]T**N**E**CG**YA	1 (0.59)
		F[**R/L**]T**N**E[S/**C**][**G**/A]YA	1 (0.59)
		F[**R/L**]T**N**E[S/**C**]**G**YA	1 (0.59)
		F[**R/L**]T**N**E**CG**YA	1 (0.59)
		F[**R/L**]T**N**ESGYA	1 (0.59)
		F[**R**/S]T**N**ESAYA	1 (0.59)
		F**K**T**N**E[S/**C**][G/A]YA	2 (1.2)
		F**K**T**N**E[S/**C**]GYA	1 (0.59)
		F-T**N**E[S/**C**][G/A]YA	1 (0.59)
		F-T**N**ESAYA	1 (0.59)
		F-T**N**ESGYA	1 (0.59)
		**LR**TSESAYA	1 (0.59)
		- - -**N**E[S/**C**][G/A]YA	1 (0.59)
		- - -**N**ESGYA	1 (0.59)

PvDHPS/DHFR/ MDR1	DHFR 57/58/61/117 DHPS 380/382/383/385/553 MDR1 976	F**K**T**N**E**CG**YAY	53 (31.2)
		F**R**T**N**E**CG**YAY	27 (15.9)
		F**R**T**N**ESAYAY	21 (12.4)
		F**K**T**N**ESAYAY	13 (7.6)
		F**R**T**N**ES**G**YAY	14 (8.2)
		F**K**T**N**ES**G**YAY	8 (4.7)
		F**R**T**N**E**CG**YA**F**	4 (2.4)
		F**K**T**N**E[S/**C**][**G**/A]YAY	2 (1.2)
		F**R**T**N**ESAYA**F**	2 (1.2)
		F**R**T**N**ES[**G**/A]YAY	2 (1.2)
		FST**N**ESAYAY	2 (1.2)
		FSTSESAYAY	2 (1.2)
		F[**R/K**]T**N**E**CG**YAY	1 (0.6)
		F[**R/L**]T**N**E[S/**C**][**G**/A]YAY	1 (0.6)
		F[**R/L**]T**N**E[S/**C**]**G**YA[F/Y]	1 (0.6)
		F[**R/L**]T**N**E**CG**YAY	1 (0.6)
		F[**R/L**]T**N**ES**G**YAY	1 (0.6)
		F[**R**/S]T**N**ESAYAY	1 (0.6)
		F**K**T**N**E[S/**C**]**G**YAY	1 (0.6)
		**LR**TSESAYAY	1 (0.6)
		- - -**N**E**CG**YAY	3 (1.8)
		- - -**N**ESAYAY	2 (1.2)
		F-T**N**E[S/**C**][**G**/A]YAY	1 (0.6)
		F-T**N**ESAYAY	1 (0.6)
		F-T**N**ES**G**YAY	1 (0.6)
		- - -**N**E[S/**C**][**G**/A]YAY	1 (0.6)
		- - -**N**ES**G**YAY	1 (0.6)
		- - -SESAYAY	2 (1.2)

S5 Table: Analysis of Molecular Variance of P. vivax by collection sites

Source of variation	Sum of squares	Variance componets	Percentage variation
Between populations	90.5	0.13	1.21
Within populations	1289	10.92	98.78
Total	1379.5	6.37	

p-value = 0.13

S6 Table: Analysis of Molecular Variance of P. falciparum by collection sites

Source of variation	Sum of squares	Variance componets	Percentage variation
Between populations	6.88	0	0
Within populations	41.7	1.67	100
Total	48.58	1.67	

p-value = 0.69
